# Preliminary Assessment of Microbiome Changes Following Blood-Feeding and Survivorship in the *Amblyomma americanum* Nymph-to-Adult Transition using Semiconductor Sequencing

**DOI:** 10.1371/journal.pone.0067129

**Published:** 2013-06-24

**Authors:** Arturo C. Menchaca, David K. Visi, Otto F. Strey, Pete D. Teel, Kevin Kalinowski, Michael S. Allen, Phillip C. Williamson

**Affiliations:** 1 Department of Forensic and Investigative Genetics, University of North Texas Health Science Center, Fort Worth, Texas; 2 Department of Biological Sciences, University of North Texas, Denton, Texas; 3 Department of Entomology, Texas AgriLife Research, Texas A&M University, College Station, Texas; 4 Center for Learning and Development, University of North Texas Health Science Center, Fort Worth, Texas; Argonne National Laboratory, United States of America

## Abstract

The physiology of ticks supports a diverse community of non-pathogenic and pathogenic organisms. This study aims to initially characterize the microbial community present within colony-reared *Amblyomma americanum* using PCR of the variable region 5 of the 16S rRNA gene followed by semiconductor sequencing and classification of sequence data using the Ribosomal Database Project and MG-RAST analysis tools. Comparison of amplicon library datasets revealed changes in the microbiomes in newly engorged nymphs, newly-molted adults, and aged adults, as well as ticks exposed to different environmental conditions. These preliminary data support the concept that microbe survivorship and diversity are partially dependent upon environmental variables and the sequence of blood feeding, molting, and aging. The maintenance and/or emergence of pathogens in ticks may be dependent in part on temporal changes in the microbial community of the tick microbiome.

## Introduction

The lone star tick, *Amblyomma americanum*, is one of the most commonly encountered ticks in the eastern United States [1]. It can be found from Texas to Oklahoma and Missouri, extending eastward throughout the southern U.S. with recent expansion into New England as far north as Maine [2]. It is second only to the brown dog tick, *Rhipicephalus sanguineus*, in infesting human residences in the Southern Region. However, in some cases *A. americanum* may dominate as suggested by one study which found that 83.0% of ticks parasitizing humans in Georgia and South Carolina were identified as lone star ticks [1]. *A. americanum* also has a wide host range and is recognized as an economic pest of livestock [2]. Over recent decades *A. americanum* has become increasingly recognized as a vector of human pathogens including *Rickettsia rickettsii*, *Ehrlichia chaffeensis*, and (putatively pathogenic) *Borrelia lonestari* [3].

*A. americanum* exhibits a three-host life cycle in which each post-embryonic stage (larvae, nymph and adult) takes a blood-meal then falls from the host as an engorged tick to molt to the next stage or to lay eggs as an adult female. The off-host periods are spent at the soil-vegetation interface where temperature and humidity determine tick molting success and survival rate. *A. americanum* overwinters as nymphs or adults, and adult ticks are known to survive more than one year in optimal vegetation habitats [4]. All questing life stages readily attack humans, with the highest incidence of tick bites occurring in the spring and summer months; however, in southern latitudes exposure to questing ticks can be year-round [4], [5]. People who are at highest risk are those who work outdoors, such as those in construction, landscaping, forestry, farming, and wildlife management, or who are exposed through recreational activities [6], [7].

Recent studies have used PCR amplification and analysis of conserved genes such as 16S rRNA to characterize the microbiome of the lone star tick [8], [9]. A study of wild-caught adult lone star ticks from the Midwest and Atlantic states found the most prevalent bacteria to be *Coxiella* and *Rickettsia*. *Arsenophonus spp.* were detected at high but variable levels. Medically important bacteria such as *Ehrlichia* and *Borrelia* were confirmed at low levels across all states tested. Co-infections of endosymbionts were described, and data revealed that 80% of ticks contained more than one endosymbiotic microbial taxon [10]. Clay *et al.* identified three vertically transmitted endosymbionts by direct probing of adult ticks. In that study, a *Coxiella* symbiont occurred with 100% frequency in the tested samples. Additionally they suggested that there exists a close phylogenetic relationship between these symbionts and known human pathogens. DNA from *Rickettsia amblyommii*, a spotted fever group *Rickettsia*, has been detected in the lone star tick at a rate of up to 80.5% in pooled samples [9]. Small scale clone libraries of the internal tissues of *A. americanum* have been studied using a wide variety of primer sets including 16S rDNA and various genus and species specific primers that also identified large numbers of the genus *Coxiella* (89%) in colony-reared ticks [11]. Small amounts of *Rickettsia* spp. were found in wild-caught pre-fed ticks (2.2%), but post-feeding increased the number to over 46.8%. These *Rickettsia spp.* were found to be similar to the spotted fever group *Rickettsia*, including *R. ambylommii* and *R. massilae*.

Nymphal *A. americanum* depend upon a single feeding of whole-blood to provide nourishment through metamorphosis to adult ticks, and to sustain the succeeding unfed adults until they locate and attach to the next host (a period that can exceed one year) [4]. No other nourishment is taken during the off-host period. The blood meal is condensed by the removal of water and retained in the tick mid-gut where it undergoes hemolysis and intracellular digestion [12]–[14]. The principle component for digestion is globin, while the heme moiety is rejected as waste. The mid-gut not only serves as the vessel for digestion, but also for nutrient storage. The mid-gut is of endodermal origin, thus during molting the mid-gut makes the transition from nymph to adult tick intact. This transtadial linkage is considered important for the survivorship and vertical transmission of pathogens [13]–[15]. In this study, we conduct a longitudinal experiment to test the hypothesis that the microbiome of *A. americanum* changes over the transition from fed nymph, to newly-emerged adults, to aged adult ticks, and explore effects of environmental exposure on survivorship and bacterial diversity.

## Materials And Methods

### Ethics Statement

Ticks used in this study were from a colony of *A. americanum* maintained at the Tick Research Laboratory, Texas AgriLife Research, Texas A&M University, College Station, TX, using chickens as hosts for larvae and nymphs, and cattle as hosts for adults as approved under Animal Use Protocol No. 2011-213, Institutional Animal Care and Use Committee (IACUC) LACC, Office of Research Compliance, Texas A&M University, College Station, TX 77845-1186. This colony originated from field collections of ticks in Edwards and Sutton Counties. A random sample of 67 adult ticks from this colony were screened by PCR in a genus-specific manner for the presence of *Borrelia*, *Ehrlichia*, and *Rickettsia* and demonstrated to be undetectable by these methods [16].

*A. americanum* nymphs were fed on a single chicken (*Gallus gallus*) to provide a cohort of fed nymphs ensuring a common source of blood with common nutritional and non-nutritional constituents for all ticks in the experiment. Ticks were incubated for molting to adults starting in November 2011 until March 2012. The chicken for this study came from a commercial poultry operation and was a healthy bird of good plumage, weight and dietary intake. The bird was fed a commercially available poultry ration designed for maintenance and without antibiotics. The newly engorged nymphs provided an estimation of the diversity and relative abundance of microbes associated with the newly blood-fed ticks. Changes to the internal tick microbiome relative to life stage, environmental conditions and age were assessed using PCR targeted to the V5 region of the 16S rRNA gene, followed by massively parallel sequencing of the reaction products. Microbial diversity across the life stage transition from fed nymph to molted adult, and aged adult ticks in controlled and environmentally natural conditions were monitored. Table 1 summarizes the project time-line and defines subsets of ticks randomly selected to comprise 6 treatment groups and one control group. Upon collection, the specimens in each treatment group and control were killed and maintained at −80°C until DNA extraction was performed. Daily environmental conditions for the month of December 2011 and January through March 2012 are accessible online (http://climatexas.tamu.edu/index.php/monthly-reports/college-station-monthly-summaries).

**Table 1:** Project time-line and identification of experimental treatments to assess the microbiome of the lone star tick,*Amblyomma americanum*, from nymphal blood feeding, separation of cohorts into environmental conditions, and aging of adult ticks.

Project Day	Project/Event Observation	Treatment Group Identification
**0**	Chicken infested with nymphs	
**6**	Blood-engorged nymphs drop from chicken. Two cohorts of nymphs were established: one placed into incubator (30°C/88% RH) constant conditions, and the other confined in screened chambers placed outdoors under canopy exposed to daily environmental cycles.	**Nymph** = subset of 10 newly engorged nymphs.
**35**	Adult tick emergence in incubator, cohort complete.	**NM-I** = subset of 10 newly molted adults from incubator cohort.
**87**	Ageing adult ticks in incubator and outdoor environments assessed for mortality and subsampled.	**NM-O** = subset of 10 newly molted adults from outdoor cohort. **AA-I** = subset of 10 incubator aged adults (60 days). **AA-I-NSC** = subset of 10 incubator aged adults (60 days).
**127**	Ageing adult ticks in incubator and outdoor environments assessed for mortality and subsampled.	**AA-O** = subset of 8 outdoor aged adults (48–65 days).**AA+-I** = subset of 9 incubator aged adults (100 days).

Treatment group sample subsets were randomly chosen and frozen at −80°C. NSC, Non-sterilized Surface Control group; RH, relative humidity.

### Extraction of DNA from Tick Samples

To prepare the ticks for total DNA extraction, specimens were surface sterilized by placing them in 0.525% sodium hypochlorite solution, followed by rinsing in ddH_2_O, followed by rinsing with 100% EtOH, then allowing specimens to air dry. This step was not performed with the non-sterilized control group (AA-I-NSC). After surface sterilization, the ticks were bisected vertically with a sterile scalpel and the entire tick was placed into a 2ml screw top test tube containing sterile 2.4 mm ceramic beads. The entire tick was used for total DNA extraction using the E.Z.N.A Mollusc DNA kit (Omega Bio-Tek Inc., Norcross GA) following the manufacturer’s protocol with the following minor modification. To crush the ticks during the initial extraction process, specimens were vortexed with the 2.4 mm ceramic beads for 10 minutes at maximum speed. The manufacturer’s protocol was followed, resulting in two elutions producing 170 µl of DNA sample extracted from the whole tick. The DNA extract was stored at −20°C until future use. DNA extraction, pre-PCR and post-PCR handling of samples were performed in separate locations to prevent cross contamination. In parallel with tick samples, an extraction reagent blank was carried though the process to monitor for contamination. PCR products were gel purified and combined according to groups prior to sequencing.

### 16S rDNA PCR Amplification

PCR amplification of the variable five (V5) region of the 16S rDNA was performed in a nested fashion for each tick DNA sample as described by Visi *et al* [17]. The initial amplification used primers 27F and 1492R that target a large 1.5 Kb section of the 16S rRNA gene [18]. A PCR master mix solution was created containing the following per 50-µl PCR reaction: 33 µl ddH_2_O, 10 µl 5X PCR buffer, 0.5 µl dNTPs (25 mM each), 2.5 µl 5-µM forward primer, 2.5 µl 5-µM reverse primer, 0.5 µl Herculase II Fusion DNA polymerase (Agilent Technologies Inc., Santa Clara CA) and 1 µl of input template DNA from each extracted whole tick DNA sample. A PCR reagent blank was run with this amplification along with testing the DNA extraction reagent blank. PCR was performed using a Bio Rad MJ Mini 48-well thermal cycler with 0.2-ml PCR tubes. The PCR cycling conditions were as follows: an initial denaturation step at 95°C for 5 min, followed by 24 cycles of denaturation at 95°C for 30 s, primer annealing at 63°C for 30 s, and extension at 72°C for 45 s, and followed by a final extension at 72°C for 5 min. After amplification, the PCR products were purified using an Agencourt AMPure®XP (Beckman Coulter Inc., Brea CA) PCR purification system and the final purified products were eluted into a final volume of 20 µl 1X tris EDTA buffer, pH 8.0.

### Nested PCR Amplification

The nested PCR targets the variable 5 (V5) region located within the 16S rDNA amplicon. This PCR primer set includes additional sequences that are required by the Ion Torrent system: forward primer Ion P1-E786F (Ion Torrent specific regions for each of the primers are shown in bold) 5′**CCATCTCATCCCTGCGTGTCTCCGACTCAG**GATTAGATACCTGGTAG and reverse primer IonA-E939R 5′**CCTCTCTATGGGCAGTCGGTAGT**CTTGTGCGGGCCCCCGTCAATTC. For each PCR, the following constituents were added: 30.5 µl ddH_2_O, 10 µl 5X PCR Buffer, 0.5 µl dNTPs (25 mM each), 2.5 µl 5 µM forward primer, 2.5 µl 5 µM reverse primer, 1.5 µl DMSO 100%, 0.5 µl Herculase II Fusion polymerase and 2 µl of purified 16S amplicon product. The PCR cycling conditions were as follows: an initial denaturation step at 98°C 3 min, followed by 40 cycles of denaturation at 98°C 15 s and primer annealing and extension at 61°C 15 s. Amplification cycles were followed by a single final extension step at 72°C for 5 min. The reaction products were purified using the same magnetic bead DNA PCR amplicon purification kit (Agencourt AMPure®XP PCR purification system) following the manufacturer’s protocol. Amplicons from each tick were pooled by group in equimolar amounts and diluted to the appropriate final concentration as outlined by Ion Torrent recommendations (8.4 nM) and placed through the workflow. Emulsion PCR was performed on the OneTouch system as per manufacturer’s instruction. The resultant beads were enriched on the Ion Torrent ES prior to sequencing on the Personal Genome Machine. All samples were run independently, each on a separate 314 chip using 100-bp chemistry.

### Analysis of Sequence Data

The raw data sequencing files in standard flowgram format (.sff) were processed into FASTA and quality files using the Galaxy online bioinformatics tools (http://usegalaxy.org/) [19]. These files were then imported into the Ribosomal Database Project (RDP) pyrosequencing pipeline (Release 10, update 28 January 12, 2012) online tool (http://pyro.cme.msu.edu/), using the initial processing step [20]. This tool removes primers and low quality sequencing files, leaving the remaining high quality sequences ready for classification. The filter parameters were left to the default setting, except the minimum read length value was set to 100 bp and the reverse primer max edit distance was set to 2. The trimmed and filtered FASTA file for each sample group was then submitted to the RDP 16S Classifier used to assign 16S rDNA sequences to a phylogenetically consistent higher-order bacterial taxonomy. Taxa assignments are based on the RDP naïve Bayesian rDNA classifier algorithm. The bootstrap confidence estimate was set at 50% as recommended for sequences shorter than 250 bp [20]. RDP Classifier can rapidly and accurately provide assignments from the domain to genus level and shows that using variable 16S regions provide a good measure of biodiversity [21], [22]. Complementary analyses were performed using the MG-RAST pipeline employing both the RDP and Greengenes datasets (http://metagenomics.anl.gov/) [23]. The resultant data were imported into an excel spreadsheet where taxa were grouped and abundance profile graphs were generated for comparison. Alpha-diversity was estimated using MG-RAST metagenomic analysis server using the same sequencing reads for classification [23]. This numerical value summarizes the diversity within a sample estimated from the distribution of species-level annotations (defined as 0.03% divergence) [23]. Heat maps were also derived using MG-RAST and the same sequencing reads for classification. Results are based on Bray-Curtis distance and ward clustering. Data were normalized based on MG-RAST version 3.0. Sequences obtained during this study were deposited for public access in the MG-RAST server under the accession numbers: 4514302.3, 4514303.3, 4514304.3, 4514305.3, 4514306.3, 4514307.3, and 4514308.3.

## Results

Engorged nymphs retained in constant environmental conditions (30°C/88% RH) of the incubator (non-stressed) all successfully molted to adults in 29 days (Project day 35, Table 1). Adult ticks from these nymphs were 100 days of age (post-molt) by project day 127 with 0% mortality. Engorged nymphs retained in the outdoor environmental conditions (stressed) experienced a prolonged period for molting to the adult stage. The first individuals molted on 6 January (65 days exposure), 50% of individuals molted by 17 January (76 days exposure), and 85% molted by 23 January (82 days exposure; Project day 87, Table 1) with 15% mortality. Adult ticks from these nymphs were 48–65 days of age (post-molt) by the final project day (127; 3 March).

The number of sequencing reads per treatment group obtained from the Ion Torrent PGM analyzer ranged from 73,651 to 413,272. After using the online bioinformatic tools (usegalaxy.org and rdp.cme.msu.edu) to process the sequencing files, the number of remaining sequences used in final data analysis ranged from 16,930 to 214,883, with an average length of 112 bp (Table 2).

**Table 2:** Summary statistics of all runs on the Ion Torrent PGM.

	Pre-Processed			Post Processed		
Sample Name	Total Bases (Mb)	Average Read Lengths	Reads	Total Bases (Mb)	Average Read Lengths	Reads
**NYMPH**	52.92	147	359,218	10.49	112±1	93,544
**NM-I**	43.28	140	309,559	11.67	112±1	103,995
**NM-O**	50.95	140	363,477	20.02	112±2	177,765
**AA-I**	56.7	137	413,272	24.11	112±2	214,883
**AA-O**	9.96	135	73,651	1.8	112±1	16,930
**AA+-I**	18.82	135	139,260	3.2	111±0	28,272
**AA-I-NSC**	23.05	141	164,041	2.0	112±1	17,754

Table below shows the pre-processed and post-processed data that were analyzed.

**Figure 1:**
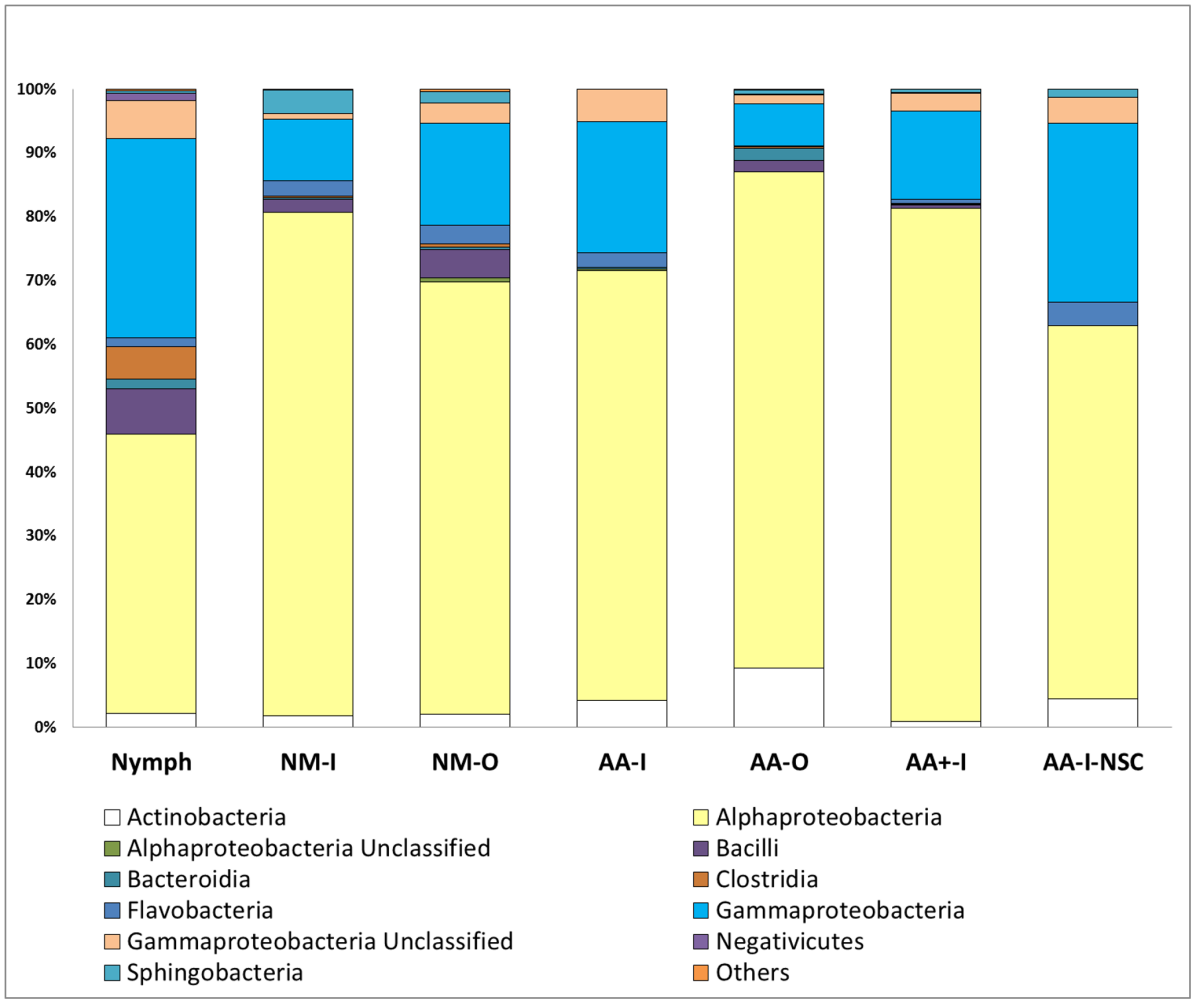
Relative Abundances of the Bacterial Classes Detected by V5 rRNA Amplicon Sequencing for Seven Tick Groups. Shows relative abundances of all samples tested at the Class taxonomic level. All Classes that had less than 0.5% contribution were pooled together into one group known as “Other”.

The bacterial composition at the class level for seven sequence datasets showed Alphaproteobacteria as the dominant bacterial class in all groups ranging from 43.8 to 80.4% (Figure 1). The engorged nymphs (Nymph) had the lowest percent abundance of Alphaproteobacteria (43.8%) and the highest level of Gammaproteobacteria (31.2%) as compared to all other sampling sets. In addition, nymphs had an increased abundance of Clostridia (5.0%) as compared with the adult groups (0%–0.5%).

### Changes in Bacterial Composition of Nymph vs. Adult Newly Molted and Incubated Ticks

**Figure 2:**
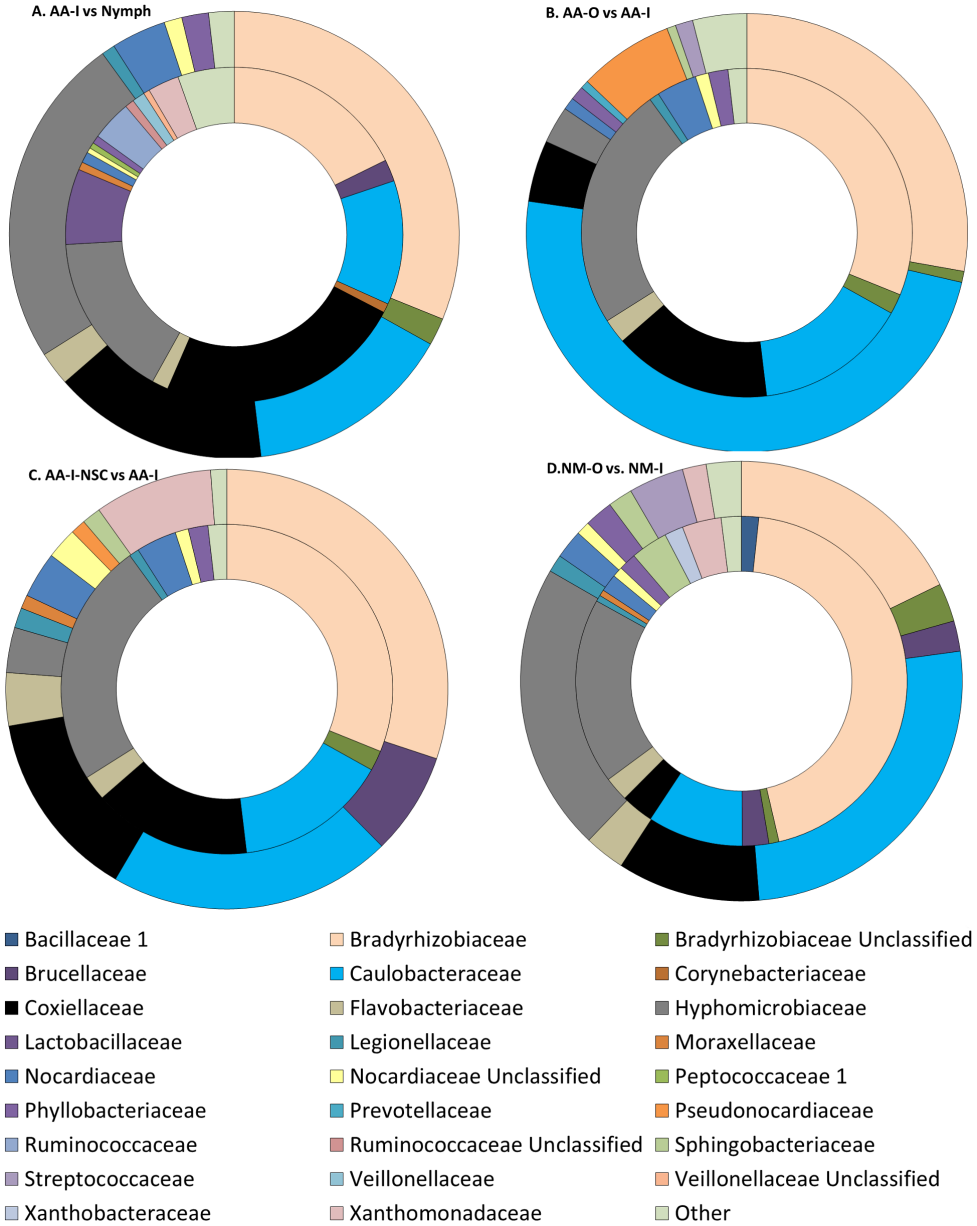
Pair-wise Comparison at the Family Taxonomic Level. **A** Classification of percent abundance detected per group of newly engorged nymphs (inner ring) and adult 60 day indoor, AA-I (outer ring). **B** Adult 60 day outdoor – AA-O (outer ring) and adult 60 day indoor – AA-I (inner ring). **C.** Comparing the non-surface sterilized 60 day aged indoor tick - AA-I-NSC (outer ring) and the 60 day aged indoor tick – AA. **D.** Newly molted outdoor (NM-O) shown on the outer ring and newly molted indoor (NM-I).

Determining changes that occurred from the Nymph stages to the adult indoor reared ticks (AA-I) showed marked contrast at the 60–day mark (Figure 2A). Specifically the largest change occurred in the increase of the family Bradyrhizobiaceae from 17.7% in Nymph to 31.2% in AA-I. Additionally, Coxiellaceae had the opposite trend in that there was a reduction from 23.8% in the blood-engorged Nymph to 15.5% in AA-I.

### Adults Ticks Aged Indoors (AA-I) vs. Adults Aged Outdoors (AA-O)

Experiments were conducted to examine if the relative changes in percent abundance of microbes at the family taxonomic level are different between adult ticks aged for 60 days (AA-I) under constant incubator conditions (30°C/88%RH) vs. ticks subjected to natural conditions (e.g. diurnal temperature variation, fluctuating humidity) for 48–60 days (AA-O). The most striking difference at the family taxonomic level (Figure 2B) is the increased percent abundance of Caulobacteraceae in outdoor-reared samples (48.7% for AA-O, vs. 15% AA-I) at the apparent expense of Hyphomicrobiaceae (3% for AA-O, vs. 24% AA-I%) and Coxiellaceae (15.5% for AA-I, 4.5% for AA-O). The AA-O ticks at this age have experienced longer molting periods and aging with mean daily maximum and minimum temperature fluctuations of 5°C, 7.9°C, 4.9°C, and 8.2°C in November, December, January and February, respectively (http://climatexas.tamu.edu/index.php/monthly-reports/college-station-monthly-summaries).

### Surface Sterilization and its Effects on Microbiome Estimation

The non-sterilized control (AA-I-NSC) group was found to include the class Sphingobacteria as well as increased relative percent abundances of Gammaproteobacteria and Flavobacteria, but decreased amounts of Alphaproteobacteria when compared to AA-I–a duplicate set of aged adult ticks which underwent surface decontamination (Figure 1). Additionally the differences between the two groups are shown to be even more substantial at the family level (Figure 2C). At this level both AA-I-NSC and AA-I had Bradyrhizobiaceae as the most abundant organisms, (31.2 and 30.1%, respectively), but there was a greater number of taxons present at the family level in AA-I-NSC. Specifically, a relatively large contingent of the family Xanthomonadaceae was detected in the non-sterilized ticks (8.6%) that was not detected in the sterilized control AA-I. Sphingobacteriaceae were similarly represented with 1.4% relative abundance detected in AA-I-NSC but none detected in AA-I (Figure 2C).

### Newly Molted Incubator (NM-I) vs. Newly Molted Outdoor (NM-O) Ticks

As shown in Figure 2D, a substantial change can be seen in the increase in the relative abundance of Caulobacteraceae (26% vs. 9%) in the newly molted outdoor group, consistent with that seen in outdoor vs. indoor aged adults. The shift was compensated for here by relative decreases in Bradyrhizobiaceae (45% to 18%) in the NM-O vs. NM-I groups, respectively. The average monthly minimum daily temperatures for outdoor nymphal molting in November, December, and January were 10.3°C, 6.7°C, and 6.8°C, respectively (Easterwood Field Weather Station, College Station, TX; Office of the Texas State Climatologist). This period included eight days in which temperatures were at or just below freezing.

### Tracking Bacterial Diversity of Incubator-Raised Ticks from Newly Molted Stage to 100 Days Old

**Figure 3:**
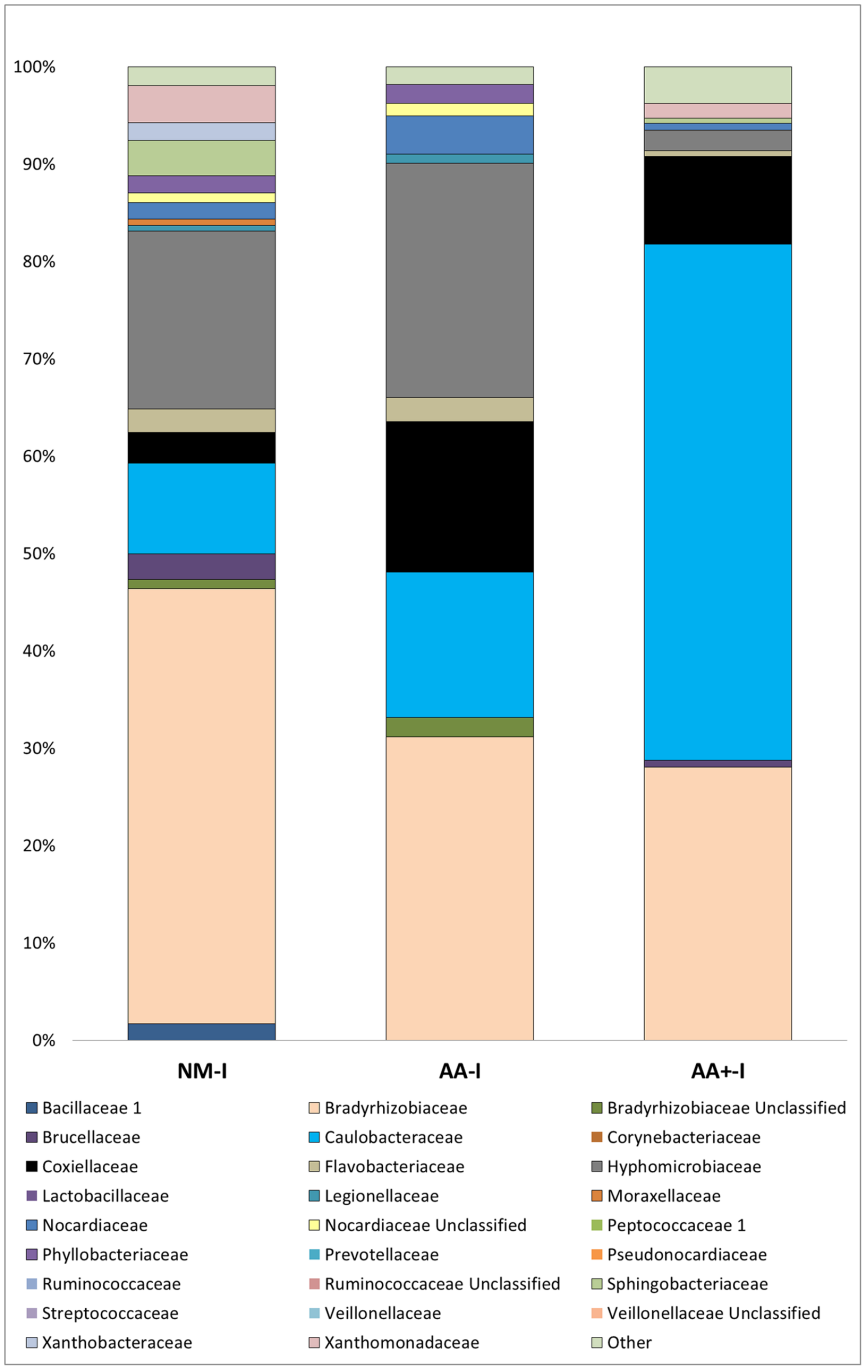
Classification counts shown as a percentage of sequences detected per group between NM-1, AA-I, and AA+-I denoting the time course change in indoor-reared ticks at the Family taxonomic level.

To investigate whether bacterial diversity changes occur as the ticks progressed in age, three samples were tested: newly molted indoor (NM-I), adults aged 60 days indoor (AA-I), and adults aged 100 days indoor (AA+-I). As shown by the bar graph representation of diversity abundance (Figure 3), the trend shows that as the ticks aged, the diversity collapsed into one dominant family, the Caulobacteriaceae, at the expense of members of the families Hyphomicrobiaceae and Bradyrhizobiaceae.

### Diversity Estimates and Community Comparisons

Alpha values were calculated at the species level and they are summarized in Table 3. Results indicate the highest diversities are the nymphs and newly molted adults. However, these appear to decrease in diversity over time under both growth conditions. Ticks reared outdoors were far less diverse than their indoor counterparts.

**Table 3:** Alpha diversity values for all groups at the species level calculated using MG-RAST analysis server.

Sample Data	α-Diversity
Nymph	9.65
NM-I	11.14
NM-O	8.88
AA-I	8.28
AA-O	4.52
AA+-I	3.50

Diversity declines upon aging (Groups AA-I, AA-O, and AA+-I) and with exposure to variable conditions.

**Figure 4:**
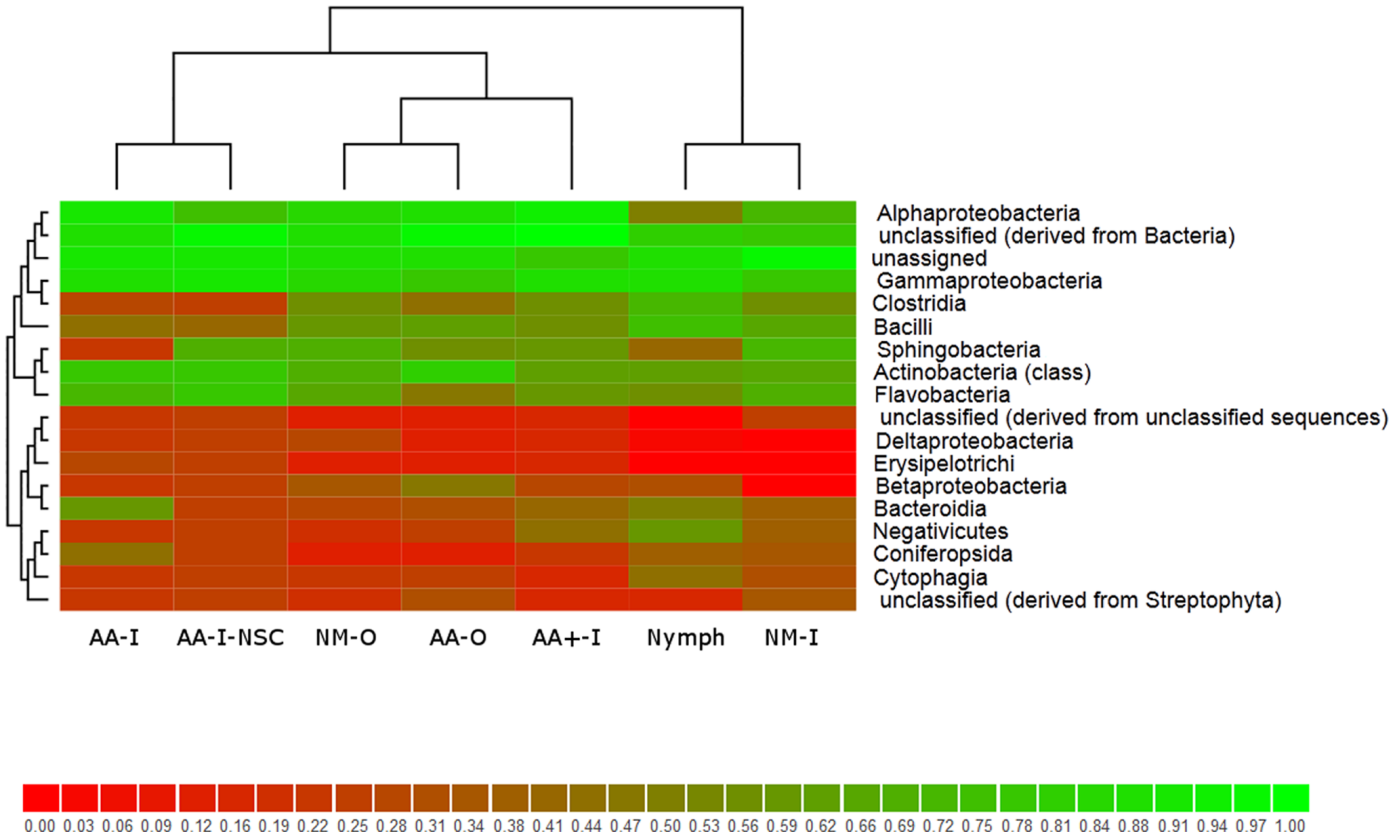
Heat map at the Class taxonomic classification of all sample groups. The dendrograms at the top show the environments relation to each other and cluster together based on similarity. The left side dendrograms shows similarity among categories or in this case the different family level organisms. The scale shows highest abundance at green versus the lower abundance on red.

A heat map was also generated for all samples at the class taxonomic level using the MG-RAST server and shown in Figure 4 (an alternative color scheme version is available in the Figure S1). The x-axis of the heat map specifically refers to the different metagenomic samples, and the y-axis as the normalized abundance of the different bacterial families. Green represents high abundance and red indicates a relative decrease in abundance or absence. The left dendrogram refers to the frequency of co-occurrence among groups in terms of whether they are present or not (e.g. when Gammaproteobacteria are present, Clostridia are likely to be present). The top dendrogram indicates the similarity between samples (e.g. tick life stage or sample type). The dendrogram results (top of Figure 4) indicated that the two indoor aged adult samples that differed only in surface sterilization treatment (AA-I and AA-I-NSC) clustered together, as did the two outside raised samples NM-O and AA-O. AA+-I, the oldest tick group, was one branch away from the outside reared set.

## Discussion

In this study, changes were observed in the diversity and abundance of the microbiome of *A. americanum* relative to life stage transition, environmental conditions and tick age, as well as identification of taxa associated with the tick’s internal and external surfaces. The 16S ribosomal gene was targeted using a set of universal primers (27F and 1492R), which amplify the bacterial population present in the tick DNA extracts. Following this initial amplification, a nested PCR for the V5 region of the 16S ribosomal gene was successful in producing an amplicon of approximately 200 bp. To monitor the amplifications and extractions for contamination, DNA extraction and PCR reagent blanks were carried through the amplification process and all controls performed appropriately.

Studies have shown that short sequencing reads from 16S rDNA variable regions, like those generated by pyrosequencing and next-generation sequencing platforms, can capture the microbial diversity for taxonomic classification by comparison to known ribosomal sequence databases (RDP or Greengenes) [22], [24]. Additionally, simulations have shown that primers targeting the V5 region allow excellent coverage and recovery of the microbial diversity required for a metagenomic survey for their size [21], and small amplicon metagenomic libraries like the one employed in this study can provide robust diversity assessments of organisms [25].

Additionally, we chose the Ion Torrent PGM platform due to its inherent low-cost per sequencing run that allowed us to perform the next-generation sequencing on site. Several recent papers describe using the Ion Torrent PGM for sequencing 16S amplicons [17], [26]–[28], but it remains a relatively new platform. The sequencing described here was performed at the very advent of semi-conductor sequencing and were limited to ∼100 bp runs. 200- and 400- bp reagent kits and protocols have recently been released. Application of the latter will enable use of primers and regions more commonly used in 454 pyrosequencing.

The workflow used here employed a nested PCR approach. This was adopted to eliminate production of spurious bands detected during early testing of direct amplification with the adapter-fused primers. Results described by others suggest that additional PCR bias introduced using the approach as described here is minimal [29]. However, like any PCR reaction, primer selection can introduce bias as a function of target specificity and would be expected to be additive in the nested approach. For example, widely used primers that target the 8–27 bp region (*E. coli* numbering) of the 16S region are known to exclude some phyla of bacteria (e.g. TM7 and Verrumicrobia) [30], [31]. Increased read lengths on the Ion Torrent platform will allow targeting of other regions in the future, which may obviate the need for nested PCR.

This pilot study used *G. gallus* as a host. It is understood that this could influence the microbiome however, the chicken (*G. gallus*) and 2 other gallinaceous birds (wild turkey and quail) are among at least 10 bird species previously documented as hosts for larvae and nymphs of *A. americanum* [11], [32]. Determining the extent to which chicken-fed (or any source) ticks maintain endosymbionts or pathogens was outside the scope of this study [11], [33].

Importantly, *Coxiella* endosymbionts were detected in all samples. This finding is consistent with several other reports in the literature and supports the conclusion that the data and techniques applied here were appropriate. The highest BLAST match for this group was identified as a *Coxiella* endosymbiont of *A. americanum* (data not shown). Also of interest is the observed abundance of *Bradyrhizobium spp.* This taxon is not unusual for its appearance, but has not previously been shown at such high abundances. Tick aging under variable outdoor conditions decreased the relative abundance of this taxon in the samples and suggests that its high abundance may be the direct result of the stress imposed by oscillating daily environmental conditions in contrast to constant laboratory conditions, or the feeding source (chicken) employed in this study. Nevertheless, several reports of other nitrogen-fixing Rhizobiaceae associated with a variety of ticks have been published [34]–[36], so the finding is not without precedent. The particular significance of nitrogen-fixing bacteria to the physiology of the host, however, remains to be elucidated. The *Bradyrhizobium* eventually begin to give way to members of the genus *Phenylobacterium* (family Caulobacteraceae) over time (Table 4). Little is known about this genus, with most studies describing its ability to degrade xenobiotic aromatic compounds. Of the known members of the genus, almost all are environmental bacteria. One surprising exception is the sequenced *Phenylobacterium zucineum* that was identified as a facultative intracellular bacterium infecting a human erythroleukemia cell line [37].

**Table 4:** Genus-level assignments of all sample groups.

	Nymph	NM-I	NM-O	AA-I	AA-O	AA+-I	AA-I-NSC
*Bradyrhizobium*	18%	**44%**	15%	**31%**	28%	28%	**32%**
*Chryseobacterium*	–	–	–	3%	–	–	4%
*Coxiella*	**26%**	3%	11%	17%	5%	9%	15%
*Hyphomicrobium*	17%	19%	23%	26%	3%	2%	–
*Lactobacillus*	8%	–	–	–	–	–	–
*Ochrobactrum*	–	–	2%	–	–	1%	8%
*Phenylobacterium*	11%	10%	**28%**	16%	**50%**	**54%**	21%
*Rhodococcus*	–	–	–	3%	–	–	–
*Saccharopolyspora*	–	–	–	–	7%	–	–
*Sphingobacterium*	–	4%	–	–	–	–	–
*Streptococcus*	–	–	4%	–	1%	–	–
*Xanthomonas*	3%	4%	–	–	–	2%	9%

Top six percent abundant genera are shown for each of the different *A. americanum* microbiomes. Highest percent abundant genera are shown in bold.

Also of note, previous studies by others reported detection of DNA from *R. amblyommii*, a spotted fever group *Rickettsia spp.*, in wild-caught lone star ticks at rates of up to 80.5% in pooled samples [9]. We had previously screened the tick colonies used as a source for this study for the presence of *Ehrlichia* spp., Spotted Fever Group *Rickettsia, and Borrelia* spp. by PCR (data not shown) and failed to detect the presence of these organisms’ DNA. Here, using next-generation sequencing techniques, we similarly failed to detect any of the aforementioned groups with the exception of an exceedingly small number of *Rickettsia* sp. in some but not all groups (Table 4). This finding suggests that *Rickettsia spp.* may not be obligate endosymbionts of *A. amblyomma* under our test conditions.

Overall alpha diversity was determined to be highest in the newly molted adult reared indoors (NM-I) and decreased in the aged adults (AA-I) (Table 3). This trend was also seen in the ticks reared outdoors (NM-O and AA-O), although the diversity estimates of the outdoor ticks were comparatively reduced. The loss in diversity over time could be attributed to the presence of an enhanced growth environment in the newly molted ticks or to age. The former is consistent with the findings of Heise *et al.* who reported diversity decreased over time after an initial high immediately after feeding in wild-caught *A. americanum*, independent of molting [11].

Pairwise comparison of the groups AA-I-NSC and AA-I demonstrates that surface decontamination does remove organisms, but the heat map at the family level (Figure 4) shows that these two tick bacterial compositions were highly similar with regards to diversity estimates as compared to the other groups. The results further serve as an internal control in support of our process and conclusions. Moreover, the data also suggest that certain identified taxa, including Sphingobacteraceae, Xanthomonadaceae, and Moraxellaceae, are most likely associated with the external surfaces of the tick.

The conservation of core taxonomic groups and the absence of those taxa thought to be associated with the external surface of the tick allow us to conclude that fluctuation of the observed taxa in the remaining groups can be directly attributed to internal structures of the tick, such as the midgut, hemolymph, and reproductive organs. Thus, the putative internal microbiome data for newly molted adult ticks provided an estimation of the diversity and relative abundance of microbes that were likely carried forward in the digested gut contents and other interior regions during molting and under select environmental conditions.

### Conclusions

Overall diversity declined as the ticks aged whether under continuously controlled or naturally oscillating environmental conditions. Further work is needed to determine if this is solely the result of feeding and subsequent starvation over time or if age in general is a factor. However, the data suggest that microbial populations within the tick are dynamic overall, and this study serves as a pilot for future work. Certain limitations of the present study include the inability to distinguish the microbial diversity of the individual ticks from the pooled samples, as well as identification of potential differences between male and female tick flora.

The ticks in this study were obtained from colonies reared under controlled conditions and did not contain any organisms known to be pathogenic. Given that environment seems to affect bacteria associated with the tick internal microbiome, introduction of a tick-borne pathogen under controlled conditions would provide insight into how the fluctuations of normal flora might influence the relative abundance and persistence of the pathogen. Determining the effects of environmental variables on successful growth of pathogens might further allow determination of the necessary conditions for pathogen emergence through transmission and maintenance in the vector populations.

## Supporting Information

Figure S1 **Heat map represented by** **Figure 4** **, but shown in gray scale.**
(TIF)
